# Research hotspots and trends in diabetes and insulin resistance: a bibliometric analysis

**DOI:** 10.3389/fnut.2024.1480491

**Published:** 2024-12-16

**Authors:** Shaobo Zhang, Huixin Yan, Di Cao, Weichen Sun, Jingnan Li, Jing Xu, Bailin Song, Xingquan Wu

**Affiliations:** ^1^Department of Acupuncture and Tuina, Changchun University of Chinese Medicine, Changchun, China; ^2^School of Basic Medical Sciences, Hubei University of Chinese Medicine, Wuhan, China; ^3^Department of Traditional Chinese Medicine, China-Japan Union Hospital of Jilin University, Changchun, China; ^4^Department of Tuina, Affiliated Hospital of Changchun University of Traditional Chinese Medicine, Changchun, China

**Keywords:** diabetes, insulin resistance, bibliometric, visual analysis, CiteSpace, VOSviewer

## Abstract

**Background:**

Many previous studies explored the relationship between diabetes and insulin resistance (IR); however, addressing the research gap where no bibliometric analysis had been conducted to summarize and analyze these publications, we will undertake a comprehensive bibliometric analysis to investigate the current status and emerging trends in publications examining the association between diabetes and IR.

**Methods:**

We retrieved publications related to the interaction between diabetes and IR from the Web of Science Core Collection (WoSCC). By utilizing software such as CiteSpace, VOSviewer, and Excel 2019, we analyzed and extracted relevant information from the literature to identify and delineate the research hotspots and directions in the study of diabetes and IR.

**Results:**

From 1900 to 2024, a total of 2,698 publications were included in the bibliometric analysis, showing a steady annual increase in the number of publications. The USA led in this research field, with the Harvard University being a key research institution. The author Olefsky JM, published the most papers;Defronzo RA was the most cited author. DIABETES was the journal with the highest number of published papers and was also the most cited journal. The main discipline in the field of diabetes and IR research was Endocrinology and Metabolism. The most cited article was “Mechanisms linking obesity to insulin resistance and type 2 diabetes (2006)”;“The IDF Diabetes Atlas: Global estimates of diabetes prevalence for 2017 and projections for 2045(2018)” was the most cited reference. “insulin resistance” was the most frequently occurring keyword. The main research hotspots and frontier areas in diabetes and IR research were as follows: (1) The association between IR, diabetes, and obesity was a popular research topic; (2) Cardiovascular diseases secondary to diabetes and IR were another hot topic among researchers; (3) As a core pathological change in diabetes, IR was a major therapeutic target for improving diabetes.

**Conclusion:**

This study summarized the research trends and hotspots in the field of diabetes and IR, provided valuable information and insights for scholars who focused on diabetes and IR scientific research, and offered a reference for future research directions.

## 1 Introduction

Diabetes has emerged as a global public health issue, currently affecting 463 million adults worldwide and accounting for 10% of global healthcare expenditures; the prevalence of diabetes was projected to rise to 700 million by 2045 (1–3). Diabetes was a chronic metabolic disorder characterized by persistent hyperglycemia, with its etiology involving IR and reduced insulin secretion from *β*-cells, where IR was considered a precursor to diabetes, as individuals with IR require higher levels of insulin to facilitate glucose uptake into cells (4–6). The concept of IR was initially proposed by Fatu (1931) and Himsworth (1936), and later gained widespread attention after the development of the radioimmunoassay by Yalow and Berson (7, 8). IR referred to the reduced sensitivity of insulin target tissues, such as adipose tissue, skeletal muscle, and the liver. In the early stages, pancreatic *β*-cells could compensate by increasing insulin secretion to offset the deficiency; however, over time, their function gradually deteriorated, leading to impaired glucose tolerance, prediabetes, and the onset of type 2 diabetes (9, 10). Therefore, as a primary pathogenic factor in diabetes, improving IR was crucial for the prevention of diabetes (11). To effectively improve IR, it was essential first to understand its underlying mechanisms. Although the mechanisms of IR were not yet fully elucidated, several key mechanisms had been proposed, including inflammation, oxidative stress, insulin receptor mutations, endoplasmic reticulum stress, and mitochondrial dysfunction (12). Previous bibliometric analyses have primarily focused on other complications of diabetes, such as diabetic foot, diabetic kidney disease, and gestational diabetes, which are also significant contributors to the global disease burden (13–15). However, insulin resistance, as a common precursor to many of these complications, remains a key therapeutic target. As a primary and common cause of metabolic disorders, IR is regarded as one of the core therapeutic targets for metabolic diseases, including diabetes. As a primary and common cause of metabolic disorders, IR was regarded as one of the core therapeutic targets for metabolic diseases, including diabetes. The primary therapeutic strategies for IR involved using diabetes medications (such as insulin, sulfonylureas, biguanides, and incretins) that stimulated insulin secretion or enhanced insulin sensitivity. Additionally, agents that inhibited hepatic fat synthesis and stimulate the oxidation of skeletal muscle and adipose tissue (such as adipose and anti-obesity agents) could reduce ectopic lipid accumulation, improve insulin sensitivity, and ultimately prevent or delay the onset of diabetes (16, 17). In addition to pharmacological treatments, lifestyle interventions, such as regular physical activity, a balanced diet, and weight control, have been shown to improve insulin sensitivity and are essential components of managing diabetes (18).

Bibliometrics served as a crucial quantitative measurement method for research in a specific field, facilitating the analysis and summarization of the research status, future trends, and global scientific competitiveness of a particular topic by filtering published data in databases according to research objectives and employing statistical methods as well as graphical and tabular representations (19). Despite the numerous publications on diabetes and IR within the WOSCC, there had yet to be a bibliometric and visualization analysis conducted on this specific topic. Therefore, this study aimed to review articles related to diabetes and IR published in the WOSCC from 1900 to 2024, to assist scholars and researchers in the field of endocrinology in better understanding the research hotspots and frontiers in this domain.

## 2 Methods

### 2.1 Data sources and search strategy

**Figure 1:**
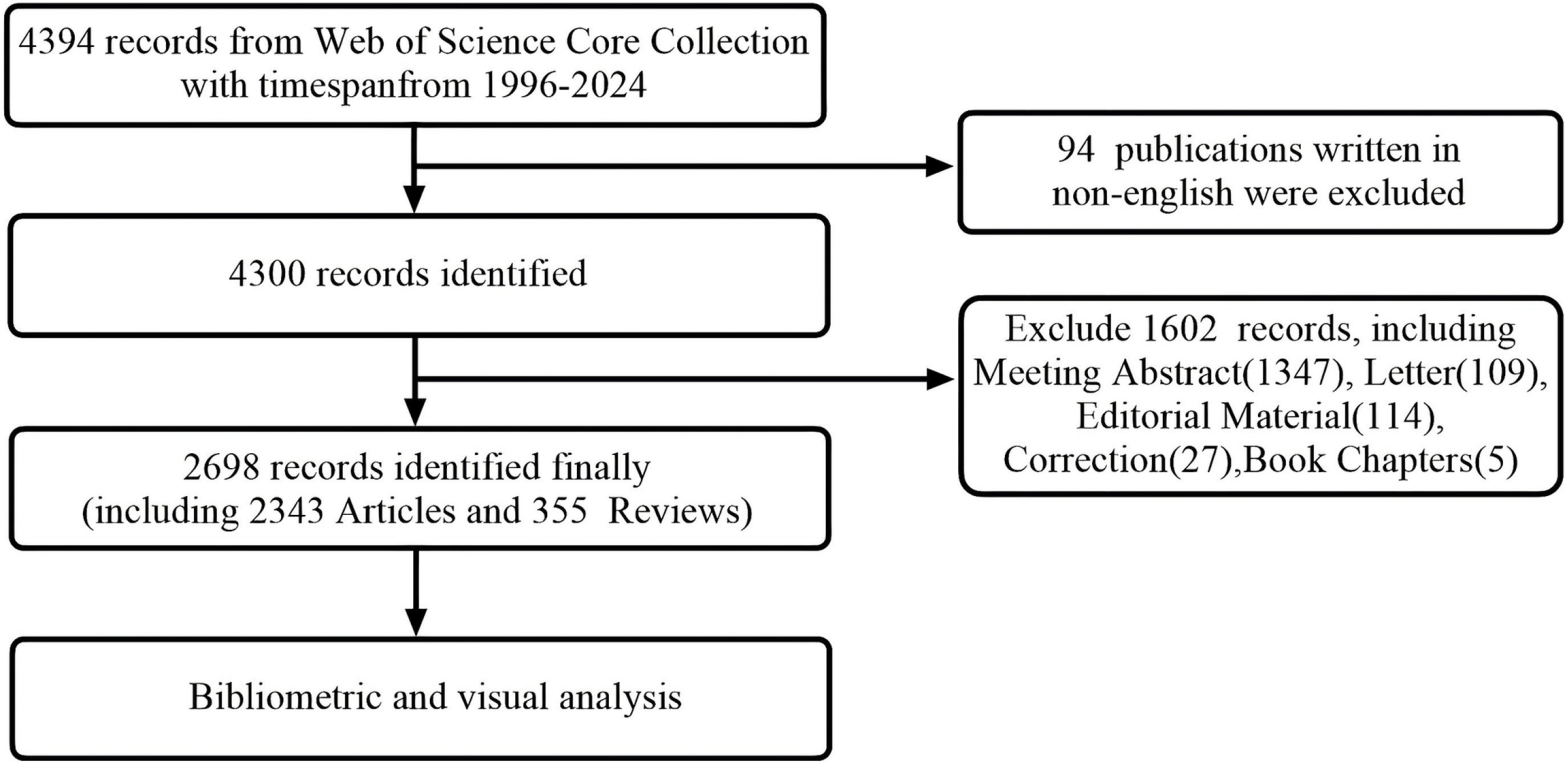
Publications screening flowchart.

In the literature search conducted by our team (Figure 1), we used the WoSCC database on July 31, 2024. The time span was set from “1900 to 2024” and the search formula was TI = (diabetes) AND TI = (insulin resistance), with the language limited to “English” and document types set to “articles” and “reviews.”

### 2.2 Data analysis

In bibliometric research, CiteSpace was one of the popular tools based on Java program information, used for literature data analysis and visualization in specific fields (20). VOSviewer was a literature analysis software tool developed by Nees Jan van Eck and Ludo Waltman from Leiden University in 2010 for creating and exploring maps based on network data (21). Depending on the research objectives and plotting requirements, we selected the appropriate software and set relevant parameters to complete the plotting and analysis. Additionally, Microsoft Office Excel 2019 was used for quantitative analysis of the publications.

## 3 Results

### 3.1 Analysis of annual publications and trends

**Figure 2:**
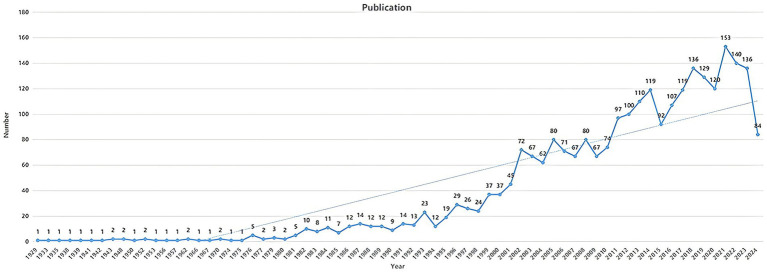
Trends in annual publications of diabetes and IR research from 1900 to 2024.

In the annual publication trend, the publication count served as an indicator of the trends and current status in a specific research field (Figure 2) (22). From 1900 to 2024, this trend reflected the research focus related to diabetes and IR. In this study, a total of 2,698 publications were retrieved, including 2,343 articles and 355 reviews. The overall trend in the annual publication volume related to diabetes and IR showed a steady increase.

### 3.2 Analysis of the trend of countries/regions, institutions, and authors

**Figure 3:**
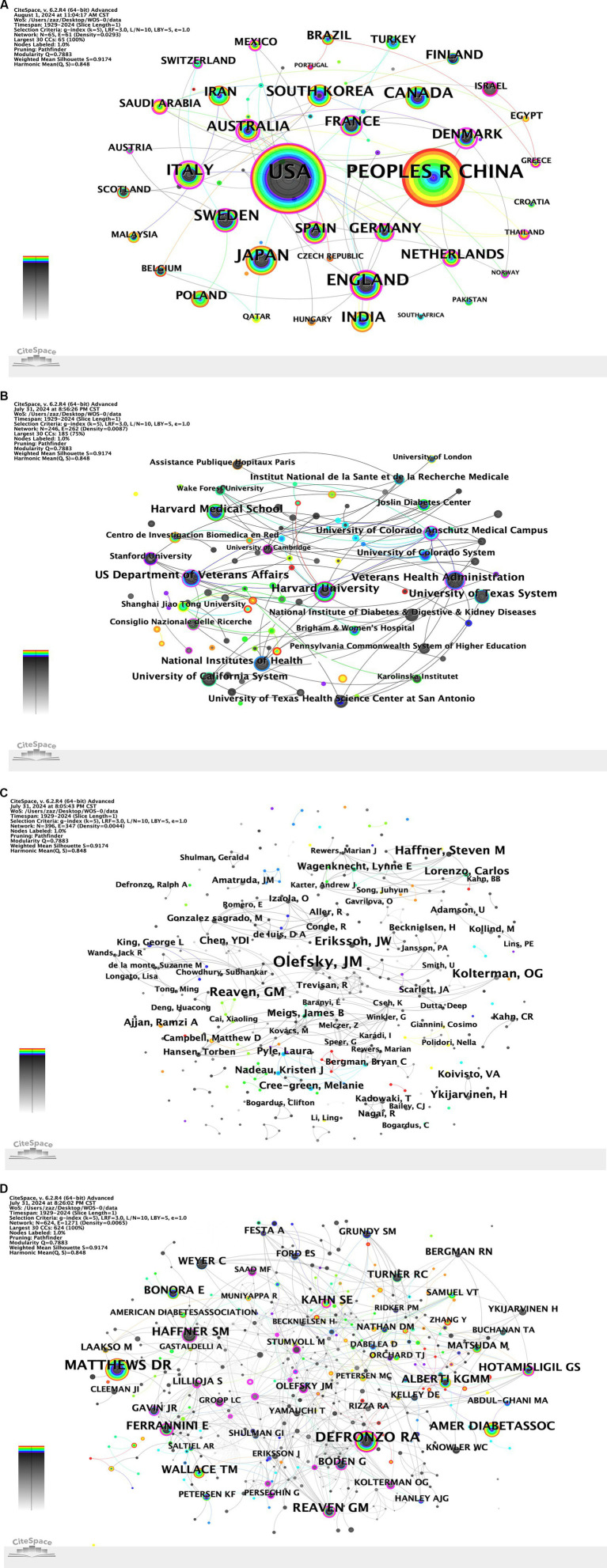
**(A)** Country co-occurrence map. **(B)** Institution co-occurrence map. **(C)** Author co-occurrence map. **(D)** Cited author co-occurrence map.

A total of 64 countries and regions contributed to the body of research on diabetes and IR. Table 1 highlighted the top 10 countries ranked by publication volume. The United States topped the list with 789 publications (25.56%), followed by China with 506 publications (16.39%). England and Japan shared the same publication count, each contributing 182 articles (5.90%). In the co-authorship network (Figure 3A), nodes represented individual countries, and the links between them indicated collaborative relationships. A country’s node size correlates with its publication output in this field, with larger nodes indicating higher productivity. The gray inner circles signified earlier publications, while the red outer circles denoted more recent work. The United States, China, Italy, Australia, and England emerged as the most prolific contributors. The centrality of a country or region reflects its significance within the network, with a positive correlation observed between collaboration and centrality. Italy boasted a centrality of 0.75, followed by Australia (0.61) and England (0.29).

**Table 1:** Top 10 countries/regions by the number of publications on diabetes and IR research.

Rank	Country/Region	Publications	Percentage (%)	Centrality
1	USA	789	25.56	0.15
2	PEOPLES R CHINA	506	16.39	0
3	ENGLAND	182	5.90	0.29
4	JAPAN	182	5.90	0
5	ITALY	127	4.11	0.75
6	CANADA	116	3.76	0
7	SWEDEN	109	3.53	0.17
8	INDIA	93	3.01	0.05
9	AUSTRALIA	84	2.72	0.61
10	SOUTH KOREA	84	2.72	0

A total of 246 academic institutions published research findings related to diabetes and IR. Table 2 listed the top 10 institutions by the number of publications. The top three institutions were Harvard University (74 papers), Harvard Medical School (63 papers), and the US Department of Veterans Affairs (62 papers). In the collaboration network (Figure 3B), the collaboration relationships and publication volumes among these institutions were illustrated, showing close connections across all institutions. The Veterans Health Administration (0.14), Harvard University (0.12), and the University of Colorado Anschutz Medical Campus (0.12) ranked as the top three in centrality, reflecting their significant roles in the collaborative network.

**Table 2:** Top 10 institutions with the most publications on diabetes and IR research.

Rank	Institutions	Country	Publications	Centrality
1	Harvard University	USA	74	0.12
2	Harvard Medical School	USA	63	0.05
3	US Department of Veterans Affairs	USA	62	0.11
4	University of Texas System	USA	57	0.03
5	Veterans Health Administration	USA	55	0.14
6	National Institutes of Health	USA	41	0.06
7	University of California System	USA	39	0.08
8	University of Colorado Anschutz Medical Campus	USA	32	0.12
9	University of Texas Health Science Center at San Antonio	USA	30	0
10	University of Colorado System	USA	28	0.01

A total of 395 authors have published papers on diabetes and IR, with their publication volumes (Figure 3C). Table 3 listed the top 10 authors and co-cited author by the number of publications or frequency. Olefsky JM published 16 papers, making Olefsky the most prolific author, followed by Reaven GM (9 papers) and Eriksson JW (8 papers). These 10 authors played significant roles in the field of diabetes and IR research. Among the top 10 authors, 7 were from the United States. The centrality of all the authors retrieved in this study was 0, indicating that collaboration among authors remained an area for improvement. The top three co-cited authors were Defronzo RA, Matthews DR, and Reaven GM. The cited author co-occurrence map (Figure 3D) presents the co-occurrence network of cited authors in the field.

**Table 3:** Top 10 authors and co-cited authors on diabetes and IR research.

Rank	Author	Publications	Co-cited author	Frequency
1	Olefsky JM	16	Defronzo, RA	619
2	Reaven GM	9	Matthews, DR	586
3	Eriksson JW	8	Reaven, GM	318
4	Haffner Steven M	8	Haffner, SM	227
5	Kolterman OG	8	Amer Diabet Assoc Professional	221
6	Lorenzo Carlos	6	Kahn, SE	197
7	Ykijarvinen H	6	Ferrannini, E	190
8	Cree-green Melanie	5	Bonora, E	175
9	Koivisto VA	5	Wallace, TM	173
10	Pyle Laura	5	Alberti, K	170

### 3.3 Journals and subjects analysis

**Figure 4:**
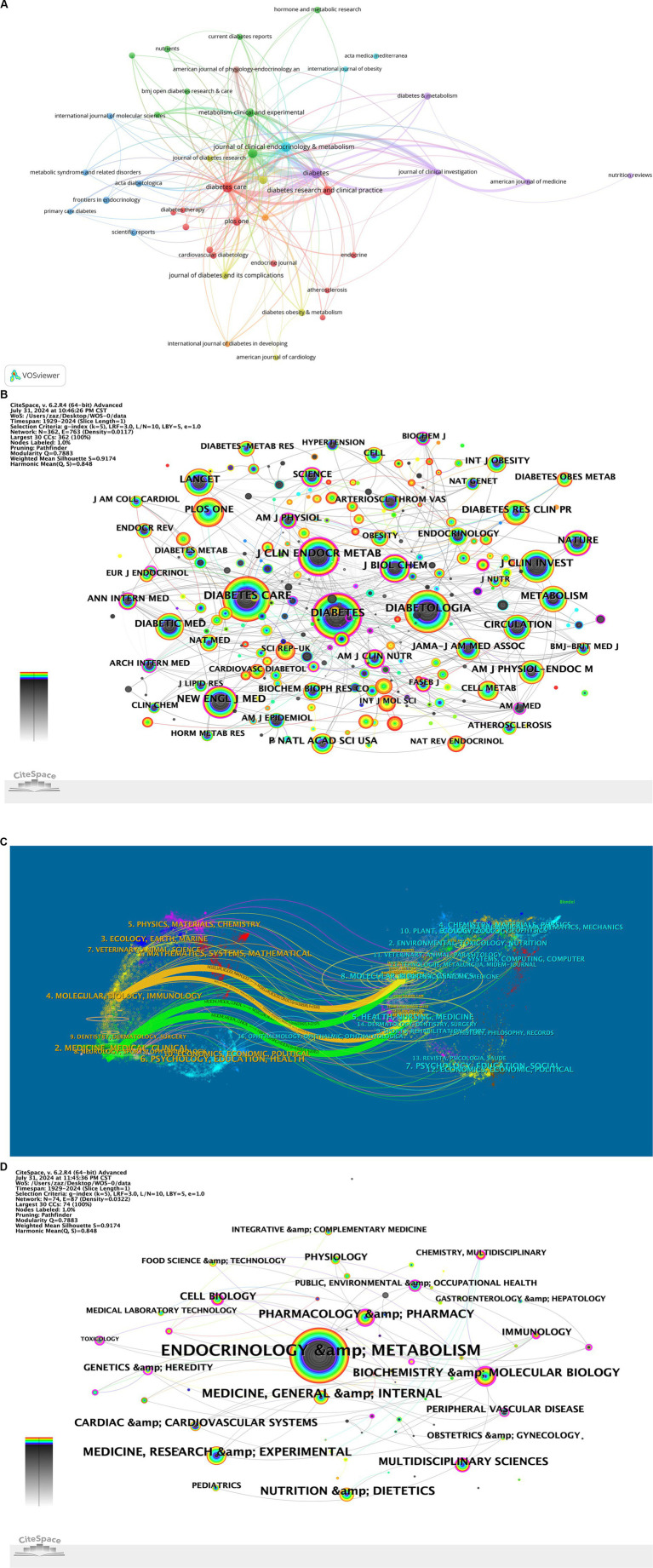
**(A)** Journal collaboration diagram. **(B)** Journal co-occurrence network. **(C)** Journal dual-map overlay. **(D)** Subject co-occurrence network.

The journals publishing articles on diabetes and IR and the collaborative relationships among them were illustrated using VOSviewer (Figure 4A). Table 4 listed the top 10 journals by the number of diabetes and IR-related publications, as well as the top 10 journals by citation frequency, including their JCR rankings and impact factors (IF). The network analysis view of the co-cited journals was shown (Figure 4B). DIABETES and DIABETES CARE ranked first and second in terms of both publication volume and citation frequency. DIABETES (Q1, IF = 6.2) had the highest number of publications (128) and was cited 2,048 times. It was followed by DIABETES CARE (Q1, IF = 14.8) with 119 publications and 1,975 citations. Among the top 10 journals by publication volume, 8 were in the Q1 quartile. DIABETES CARE had the highest impact factor among the top 10 by publication volume, at 14.8; among the highly cited journals, LANCET had the highest impact factor, at 98.4.In addition to publication volume and citation frequency, the total link strength (TLS) of the top journals was analyzed using VOSviewer, which reflects the degree of connectivity between journals in the citation network. DIABETES had the highest TLS of 744, followed by DIABETES CARE with a TLS of 639, and Journal of Clinical Endocrinology & Metabolism with a TLS of 496. These results indicate that DIABETES and DIABETES CARE not only lead in publication volume and citation frequency but are also highly interconnected with other journals, suggesting their central role in disseminating knowledge and facilitating collaboration in diabetes research.

**Table 4:** Top 10 journals and co-cited journals related to diabetes and IR research.

Rank	Journal	Publications	JCR	IF	Co-cited Journal	Citations	JCR	IF
1	DIABETES	128	Q1	6.2	DIABETES	2048	Q1	6.2
2	DIABETES CARE	119	Q1	14.8	DIABETES CARE	1975	Q1	14.8
3	JOURNAL OF CLINICAL ENDOCRINOLOGY & METABOLISM	88	Q1	5	DIABETOLOGIA	1924	Q1	8.4
4	DIABETOLOGIA	80	Q1	8.4	JOURNAL OF CLINICAL ENDOCRINOLOGY & METABOLISM	1,490	Q1	5
5	DIABETES RESEARCH AND CLINICAL PRACTICE	74	Q1	6.1	JOURNAL OF CLINICAL INVESTIGATION	1,300	Q1	13.3
6	PLOS ONE	56	Q1	2.9	NEW ENGLAND JOURNAL OF MEDICINE	1,164	Q1	96.2
7	METABOLISM-CLINICAL AND EXPERIMENTAL	53	Q1	10.8	LANCET	1,025	Q1	98.4
8	DIABETIC MEDICINE	46	Q2	3.2	METABOLISM-CLINICAL AND EXPERIMENTAL	1,001	Q1	10.8
9	JOURNAL OF DIABETES AND ITS COMPLICATIONS	35	Q3	2.9	JOURNAL OF BIOLOGICAL CHEMISTRY	859	Q2	4
10	DIABETES & METABOLISM	28	Q1	4.6	DIABETIC MEDICINE	771	Q2	3.2

The journal dual-map overlay was presented, showing the distribution of topics, with labels representing the covered subjects and colored paths indicating citation relationships (Figure 4C). The connections and collaborations between different disciplines were illustrated (Figure 4D). In the field of diabetes and IR research, the primary discipline was Endocrinology & Metabolism, which frequently collaborated closely with several other disciplines.

### 3.4 Analysis of articles and references

Table 5 listed the top 10 highly cited articles. “Mechanisms linking obesity to insulin resistance and type 2 diabetes” published in NATURE (Q1, IF = 50.5) was cited 3,616 times. This was followed by “Hypoadiponectinemia in obesity and type 2 diabetes: Close association with insulin resistance and hyperinsulinemia” published in JOURNAL OF CLINICAL ENDOCRINOLOGY & METABOLISM (Q1, IF = 5), with 2,855 citations, and “Adiponectin and adiponectin receptors in insulin resistance, diabetes, and the metabolic syndrome” published in JOURNAL OF CLINICAL INVESTIGATION (Q1, IF = 13.3), which had 2,175 citations. All of the top 10 highly cited articles belonged to Q1 journals, indicating their significant academic impact. Notably, Guilherme A’s paper titled “Adipocyte dysfunctions linking obesity to insulin resistance and type 2 diabetes,” published in the renowned journal NATURE REVIEWS MOLECULAR CELL BIOLOGY, had an exceptionally high impact factor of 81.3.

**Table 5:** Top 10 highly cited articles in diabetes and IR research.

Rank	First author	Title	Year	Citations	JCR	IF
1	Kahn SE	Mechanisms linking obesity to insulin resistance and type 2 diabetes	2006	3,616	Q1	50.5
2	Weyer C	Hypoadiponectinemia in obesity and type 2 diabetes: Close association with insulin resistance and hyperinsulinemia	2001	2,855	Q1	5
3	Kadowaki T	Adiponectin and adiponectin receptors in insulin resistance, diabetes, and the metabolic syndrome	2006	2,175	Q1	13.3
4	TODD JA	HLA-DQ-beta Gene Contributes to Susceptibility and Resistance to Insulin-Dependent Diabetes Mellitus	1987	1920	Q1	50.5
5	GuilhermeA	Adipocyte dysfunctions linking obesity to insulin resistance and type 2 diabetes	2008	1,677	Q1	81.3
6	Yang Q	Serum retinol binding protein 4 contributes to insulin resistance in obesity and type 2 diabetes	2005	1,644	Q1	50.5
7	Dandona P	Inflammation: the link between insulin resistance, obesity and diabetes	2004	1,614	Q1	13.1
8	Kahn SE	The relative contributions of insulin resistance and beta-cell dysfunction to the pathophysiology of Type 2 diabetes	2003	1,600	Q1	8.4
9	Patti ME	Coordinated reduction of genes of oxidative metabolism in humans with insulin resistance and diabetes:: Potential role of PGC1 and NRF1	2003	1,591	Q1	9.4
10	Cho H	Insulin resistance and a diabetes mellitus-like syndrome in mice lacking the protein kinase Akt2 (PKBβ)	2001	1,498	Q1	44.7

**Figure 5:**
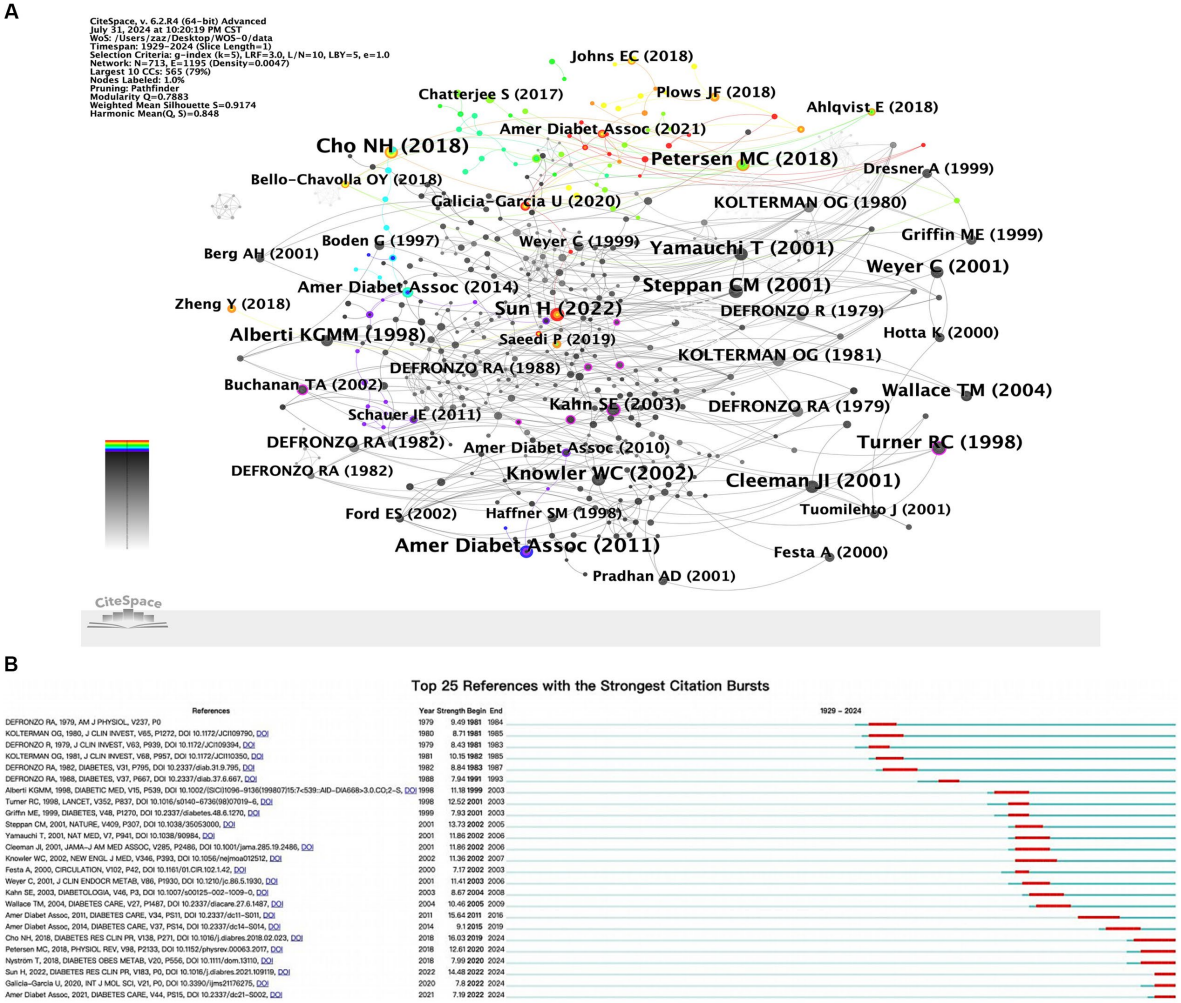
**(A)** Co-citation network map. **(B)** Top 25 references with the strongest citation bursts.

When multiple references were cited repeatedly by different documents, co-citation relationships were formed. These relationships were often used to determine the degree of association between different references (23). Table 6 listed the top 10 most frequently cited references, and the network view of the co-occurrence analysis of these references was presented (Figure 5A) “Mechanisms linking obesity to insulin resistance and type 2 diabetes” was the most cited, with 29 citations, followed by “Standards of Medical Care in Diabetes-2011 American Diabetes Association” with 27 citations. The third most frequently cited article was “The hormone resistin links obesity to diabetes,” with 26 citations. The top 25 references with the highest burst strength were shown (Figure 5B). Among them, Cho NH et al.’s article “IDF Diabetes Atlas: Global estimates of diabetes prevalence for 2017 and projections for 2045,” published in DIABETES RESEARCH AND CLINICAL PRACTICE (Strength = 16.03), was identified as the reference with the strongest citation burst.

**Table 6:** Top 10 most cited references in diabetes and IR research.

Rank	First author	Title	Year	Citations	JCR	IF
1	Cho NH	IDF Diabetes Atlas: Global estimates of diabetes prevalence for 2017 and projections for 2045	2018	29	Q1	6.1
2	Amer Diabet Assoc	Standards of Medical Care in Diabetes-2011 AMERICAN DIABETES ASSOCIATION	2011	27	Q1	14.8
3	Steppan CM	The hormone resistin links obesity to diabetes	2001	26	Q1	50.5
4	Sun H	IDF Diabetes Atlas: Global, regional and country-level diabetes prevalence estimates for 2021 and projections for 2045	2022	24	Q1	6.1
5	Cleeman JI	Executive summary of the Third Report of the National Cholesterol Education Program (NCEP) expert panel on detection, evaluation, and treatment of high blood cholesterol in adults (Adult Treatment Panel III)	2001	24	Q1	63.1
6	Knowler WC	Reduction in the incidence of type 2 diabetes with lifestyle intervention or metformin	2002	24	Q1	96.2
7	Yamauchi T	The fat-derived hormone adiponectin reverses insulin resistance associated with both lipoatrophy and obesity	2001	24	Q1	58.7
8	Turner RC	Intensive blood-glucose control with sulphonylureas or insulin compared with conventional treatment and risk of complications in patients with type 2 diabetes (UKPDS 33)	1998	22	Q1	98.4
9	Petersen MC	Mechanisms of insulin action and insulin resistance	2018	22	Q1	29.9
10	Alberti KGMM	Definition, diagnosis and classification of diabetes mellitus and its complications part 1: Diagnosis and classification of diabetes mellitus - Provisional report of a WHO consultation	1998	21	Q2	3.2

### 3.5 Analysis of keywords

#### 3.5.1 High frequency keywords analysis

**Figure 6:**
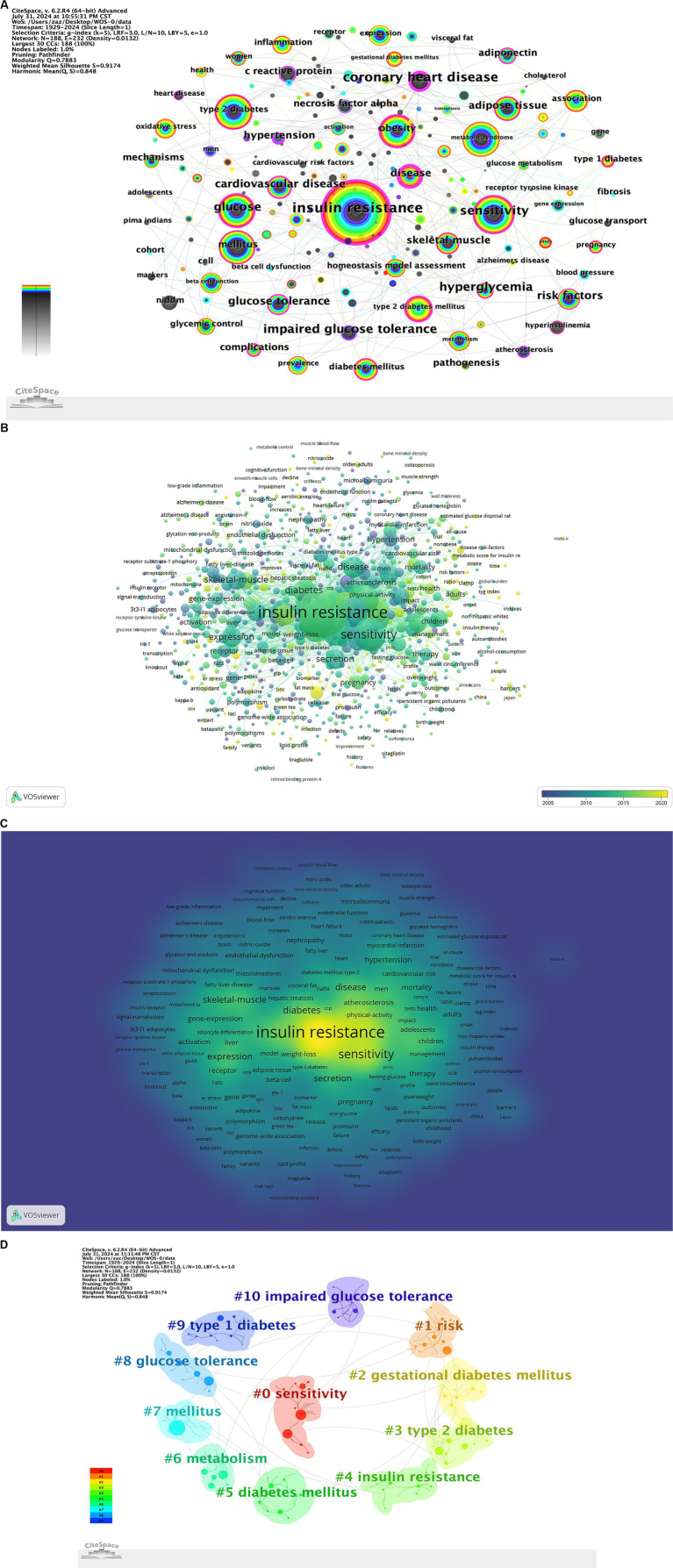
**(A)** Keyword co-occurrence map. **(B)** Keyword time zone map. **(C)** Keyword density map. **(D)** Keyword clustering map.

High frequency keywords reflected the focus of researchers over a certain period, indicating research hotspots and frontier areas (24). This study identified a total of 187 keywords. Table 7 lists the top 20 most frequently occurring keywords. The co-occurrence of these keywords was shown (Figure 6A). The keyword “insulin resistance” (*n* = 1,152, centrality: 0.54) appeared most frequently, with closely related keywords including “sensitivity” (n = 485, centrality: 0.21), “mellitus” (n = 417, centrality: 0.16), “obesity” (*n* = 413, centrality: 0.2), and “glucose” (*n* = 396, centrality: 0.3). To better understand the research hotspots and current status of diabetes and IR, we set a threshold of at least five occurrences for keywords, resulting in a keyword density map (Figure 6C). The density map shows that the main research hotspots in IR are focused on its associations with diabetes, obesity, and cardiovascular disease, as well as its biological mechanisms, clinical treatments, and impacts on specific populations.

**Table 7:** Top20 high frequency keywords in diabetes and IR research.

Rank	Key words	Frequency	Centrality	Year
1	Insulin resistance	1,152	0.54	1990
2	Sensitivity	485	0.21	1991
3	Mellitus	417	0.16	1992
4	Obesity	413	0.2	1991
5	Glucose	396	0.3	1992
6	Metabolic syndrome	360	0.02	2004
7	Type 2 diabetes	354	0.19	2000
8	Disease	196	0.63	1992
9	Association	190	0.06	1999
10	Cardiovascular disease	184	0.26	1996
11	Type 2 diabetes mellitus	179	0.28	2006
12	Skeletal muscle	178	0.1	1998
13	Adipose tissue	178	0.13	1995
14	Secretion	177	0	1993
15	Expression	171	0.04	1999
16	Glucose tolerance	171	0.27	1991
17	Prevalence	145	0.08	2004
18	Diabetes mellitus	142	0.14	1994
19	Metabolism	139	0.02	1993
20	Beta cell function	139	0.03	2002

#### 3.5.2 Keyword clustering

To understand the research frontiers of diabetes and IR since 1900, we employed CiteSpace to cluster diabetes and IR-related keywords. The keyword clustering results were shown (Figure 6D), identifying 11 clusters: #0 sensitivity, #1 risk, #2 gestational diabetes mellitus, #3 type 2 diabetes, #4 insulin resistance, #5 diabetes mellitus, #6 metabolism, #7 mellitus, #8 glucose tolerance, #9 type 1 diabetes, and #10 impaired glucose tolerance. These clusters highlight major research topics, with “sensitivity” as the largest cluster, indicating a strong research focus on insulin sensitivity. Other prominent clusters, such as “risk,” “gestational diabetes mellitus,” and “type 2 diabetes,” reflect additional critical areas of study within diabetes and IR research.

#### 3.5.3 Keyword timeline analysis

**Figure 7:**
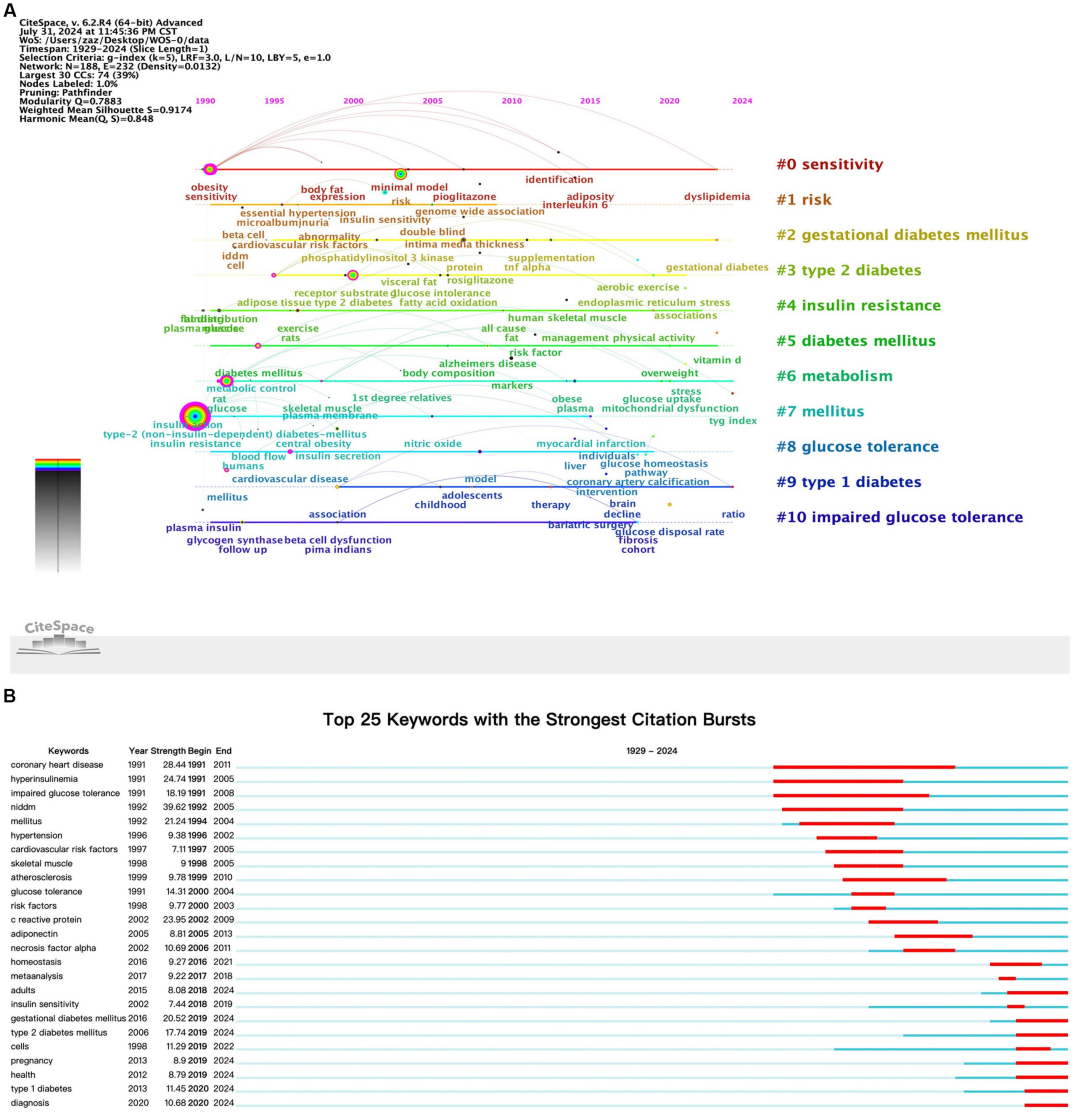
**(A)** Keyword timeline map. **(B)** Top 25 keywords with the strongest citation bursts.

The VOSviewer keyword time evolution overlay visualization was shown (Figure 6B). In the figure, the purple cluster represented the earliest keywords, the green cluster indicated earlier keywords, and the yellow cluster represented the most recent keywords. Keywords in the green cluster, such as “insulin resistance,” “sensitivity,” “skeletal muscle,” “diabetes,” and “expression,” were particularly prominent. A timeline visualization of keywords was created using CiteSpace, resulting in a clear representation of keyword trends over time (Figure 7A). The timeline view structure included the X-axis representing the publication year and the Y-axis corresponding to the keyword cluster names. Red (#0) represented the theme of “sensitivity,” which was prevalent in the earlier years of research (around the 1990s) and gradually decreased in relevance. Orange (#1) related to “risk” with keywords becoming more prominent starting in the early 2000s and continuing through to recent years. Yellow (#2) corresponded to “gestational diabetes mellitus” a theme that emerged in the 2010s and showed increasing relevance up to 2024. Green clusters (#3, #4, #5, and #6), which included themes like “type 2 diabetes,” “insulin resistance,” and “metabolism,” showed steady importance and dominated in research from the 2000s through the 2020s, indicating the sustained focus on these topics. Blue (#7 to #10) included themes like “glucose tolerance” and “type 1 diabetes,” which emerged more recently and represented growing areas of interest as research evolved. This provided a clear depiction of the stage-specific hotspots and development trajectory of keywords in diabetes and IR research over time.

#### 3.5.4 Keyword burst analysis

The emergence of keywords can indicated a sudden increase in research interest over a certain period, which may suggest future research trends. The top 25 keywords with the highest burst intensity within the research topics of diabetes and IR were presented (Figure 7B). In the figure, the blue line represented the start time of the research, while the red line indicated the duration of the keyword burst. Recently, researchers had been focusing on “diagnosis” “type 2 diabetes” “gestational diabetes” and “health.” This shift in focus highlights a growing interest in more specific aspects of diabetes and insulin resistance, particularly around diagnostics, subtype classification, and public health implications. The recent emphasis on these keywords suggests that the research community is increasingly prioritizing early detection, management strategies tailored to diabetes subtypes, and a broader consideration of overall health outcomes. This trend may indicate an evolving research landscape where prevention, personalized treatment, and population health perspectives are gaining prominence. These insights not only reflect current research priorities but may also guide future studies towards more targeted interventions and holistic approaches in diabetes and IR management.

## 4 Discussion

### 4.1 General information

Research on diabetes and IR dates back to 1929, though annual publication output remained below 10 until 1981. Starting in 1991, research articles on these topics stabilized above 10 per year, with a notable surge between 2001 and 2002, likely reflecting growing global recognition of diabetes as a major public health issue. Annual publication numbers reached 100 for the first time in 2013. Although there was a slight decline in 2015, the trend quickly rebounded, leading to a peak of 153 articles in 2021. This consistent growth indicates that diabetes and IR remain key areas of research focus, driven by advancements in diagnostic and therapeutic technologies and an increasing emphasis on metabolic health. These trends suggest that future research will likely continue to explore novel treatment approaches, prevention strategies, and the broader implications of diabetes and IR on public health.

Research on diabetes and IR was primarily concentrated in the Northern Hemisphere, with strong connections between North America, Europe, Oceania, and Asia. Oceania maintained especially close ties with North America and Europe. The United States and China had the highest publication volumes in this field, but collaboration between the two countries remained limited. Among the top 10 countries, some with a heavier burden of diabetes had lower research productivity, possibly due to a lack of funding and infrastructure. Exploring these disparities could be a valuable direction for future research. Meanwhile, China, with 114 million people with diabetes, faces significant challenges. Over the past decade, China has expanded health insurance and launched strategies to promote health and strengthen primary care. In addition, innovations in artificial intelligence and Big Data are aiding diabetes management (25). Although the U.S. and China collaborate less frequently, both have made substantial contributions to diabetes research.

The top 10 institutions by publication volume were all world-renowned universities and research institutions from the United States, closely connected to Harvard University, which demonstrated Harvard’s significant academic influence. These leading institutions included universities, research institutes, medical schools, national institutions, health science centers, and the Department of Veterans Affairs. Notably, due to the high prevalence of diabetes among veterans and the fact that the Department of Veterans Affairs operated the largest integrated healthcare system in the U.S., it provided extensive clinical data and abundant research resources (26, 27). Additionally, U.S. healthcare policies supported such research (28, 29). Institutions such as the National Institutes of Health (NIH) and the Centers for Disease Control and Prevention (CDC) have established extensive research networks that focus on diabetes, contributing to the prominence of US institutions in this field (30, 31). The multi-center institutional model developed in the U.S. for diabetes and IR research enabled multi-dimensional studies and exploration of diabetes, IR, and related diseases, making significant contributions to the advancement of this field.

Co-citation analysis identified cases where two authors’ works were cited together by a third author (Figure 3D), reflecting both their academic achievements and contributions to advancing scientific knowledge. Analysis of the most frequently cited authors in diabetes and IR research highlighted their impact and key research areas. The top three most cited authors were Defronzo RA (619 citations), Matthews DR (586 citations), and Reaven GM (318 citations).

The analysis revealed that eight of the top 10 journals were high-quality Q1-ranked, highlighting the significant scientific and clinical value of diabetes and IR research. Frequent citations in these journals underscored their critical role in advancing the field and their profound impact on subsequent research. The presence of numerous high-impact journals confirmed that insulin resistance research had become a major focus in biomedicine, particularly within Endocrinology & Metabolism, which was closely linked to disciplines like Biochemistry, Pharmacology, Cardiovascular Science, Nutrition, and Medicine.

Keyword bursts indicated a sharp increase in research interest, signaling potential future trends. Figure 7B showed the top 25 keywords with the highest burst strength in diabetes and IR research. From 1990 to 2000, keywords like “coronary heart disease,” “hyperinsulinemia,” and “impaired glucose tolerance” reflected the focus on the relationship between diabetes, IR, and metabolic diseases. From 2000 to 2010, keywords such as “C-reactive protein” and “adiponectin” highlighted research on inflammatory factors. From 2010 to 2020, keywords like “homeostasis” and “insulin sensitivity” indicated growing diversity in research topics, with a focus on systemic metabolic balance. From 2021 to 2024, emphasis shifted towards applications, as terms like “diagnosis” and “health” surged, reflecting a growing focus on clinical diagnostics and health management, alongside increased attention to cellular-level mechanistic studies.

### 4.2 Hotspots and frontiers

Based on a comprehensive analysis from multiple perspectives, including keywords, highly cited articles, highly referenced literature, and authors, the primary research trends and hot topics in the field of diabetes and IR research could be broadly categorized into three key areas.

Firstly, the relationship between IR, diabetes, and obesity has emerged as one of the key focal points of interest among researchers. Diabetes was a chronic hyperglycemic disease that occurred when the pancreas failed to produce sufficient insulin or when the body was unable to effectively utilize the insulin it produced. Type 1 diabetes mellitus was considered an autoimmune disease that developed in genetically predisposed individuals due to T-cell-mediated destruction of pancreatic *β*-cells. In contrast, type 2 diabetes mellitus (T2DM), which accounted for 90% of all diabetes cases and was the most extensively studied type, was characterized by an imbalance in blood glucose levels, with its pathogenesis rooted in IR and secretion abnormalities (32, 33). IR was a prominent characteristic of diabetes (34), serving not only as the most powerful predictor of future development of T2DM but also as a critical therapeutic target once hyperglycemia occurred (35). Defects in insulin signaling could lead to IR in key insulin target organs, including muscle, liver, and adipose tissue (36). β-cell dysfunction, inflammation, and adipocyte dysfunction were the key factors that linked obesity to IR and diabetes (37–39). Obesity were closely associated with an increased risk of IR and the development of T2DM. In obese individuals, adipose tissue released elevated levels of non-esterified fatty acids, glycerol, hormones, and pro-inflammatory cytokines, all of which contributed to the development of IR. When IR was coupled with dysfunction of pancreatic β-cells, the cells responsible for insulin secretion, it led to the onset and progression of diabetes (37). In the context of insulin resistance associated with obesity and T2DM, chronic nutritional excess leads to oxidative stress and inflammatory changes. Adipose tissue, beyond serving as a fat reservoir, was demonstrated to function as an endocrine organ that produced inflammatory cytokines. The increased concentrations of TNF-*α* and IL-6 could interfere with the anti-inflammatory effects of insulin by inhibiting insulin signaling, potentially exacerbating the inflammatory response (38, 40). Additionally, gestational diabetes mellitus (GDM) has emerged as a key area of interest due to its close association with insulin resistance, particularly in the context of obesity (41). GDM occurs when the body’s insulin resistance increases during pregnancy, often in women who are already predisposed to IR (42). It is not only a predictor of future T2DM in affected women but also represents a significant metabolic stressor during pregnancy, contributing to complications for both mother and child (43). The elevated risk of developing T2DM after experiencing GDM highlights the long-term metabolic consequences of insulin resistance during pregnancy (44). In summary, obesity played a crucial role in the development of IR (40), which was one of the most typical and common pathological changes observed in diabetes (36).

Secondly, cardiovascular diseases secondary to diabetes and IR have emerged as another focal point of interest among researchers. IR in diabetes was recognized as one of the risk factors for cardiovascular diseases and was closely associated with conditions such as coronary heart disease, hypertension, and atherosclerosis. T2DM was fundamentally a vascular disease, commonly associated with hypertension, macrovascular events, and microvascular complications. It often progressed to hypertension, which was a major determinant of cardiovascular morbidity and mortality in this patient population (45). When IR increased, the risk of coronary heart disease in non-diabetic individuals was higher than in those with diabetes, indicating that IR posed a greater risk for coronary heart disease than T2DM itself (46). IR, blood glucose levels, and inflammatory cytokines were significant risk factors for cardiovascular events in patients with diabetes complicated by coronary heart disease. Additionally, inflammation mediated by hyperglycemia and IR was considered a significant risk factor in the development of atherosclerosis, with C-reactive protein (CRP) often serving as a predictor of cardiovascular events (47, 48). Serum adiponectin levels, IR, and inflammatory cytokines were closely linked to diabetes complicated by coronary heart disease. Factors such as reduced insulin sensitivity, diminished blood pressure regulation, dysfunction of the fibrinolytic system, and heightened inflammatory responses could all affect the severity of vascular endothelial injury (47, 49). IR also played a crucial role in the pathogenesis and progression of target organ damage induced by hypertension, such as left ventricular hypertrophy, atherosclerosis, and chronic kidney disease (50). Therefore, focusing on the treatment of diabetes and IR was fundamental and essential for the prevention and management of cardiovascular diseases.

Thirdly, as the central pathological change in diabetes, IR was a primary therapeutic target in the treatment of patients with diabetes. Obesity and lack of physical activity were the main contributing factors to IR (51). The treatment strategies for IR primarily involved inhibiting fat synthesis in the liver, stimulating fat oxidation, and increasing muscle mass. These approaches aimed to reduce ectopic lipid accumulation, enhance insulin sensitivity, and ultimately prevent or delay the onset and progression of T2DM (16). Non-pharmacological management, including exercise, dietary changes, and behavioral modification, also plays a key role in managing insulin resistance and diabetes (52). Regular physical activity helps with weight reduction, improves skeletal muscle mass and VO2peak, and enhances insulin sensitivity (53–55). Additionally, dietary adjustments and behavioral strategies contribute to lowering HbA1c levels and improving overall metabolic control (56). Following these lifestyle interventions, pharmacological treatments are often necessary to further target the underlying mechanisms of insulin resistance and improve glycemic control. A variety of drugs were employed in the prevention and treatment of IR, including insulin, sulfonylureas (such as carbutamide and tolbutamide), biguanides (such as metformin, phenformin, and buformin), thiazolidinediones, incretins, sodium/glucose co-transporter-2 inhibitors, and adipose and anti-obesity agents aimed at reducing weight (17). These drugs worked through different mechanisms to improve insulin sensitivity and regulate blood glucose levels. Insulin facilitated glucose uptake into cells, while sulfonylureas stimulated pancreatic beta cells to increase insulin secretion (57, 58). Biguanides reduced hepatic glucose production and enhanced insulin sensitivity in peripheral tissues (59). Thiazolidinediones activated PPAR-*γ* receptors, promoting glucose uptake in adipocytes and reducing inflammation (60). Incretins, such as GLP-1 receptor agonists, increased insulin secretion and suppressed glucagon, thus improving postprandial glucose control (61). SGLT-2 inhibitors decreased glucose reabsorption in the kidneys, leading to increased glucose excretion in the urine (62). Additionally, anti-obesity agents targeted body weight reduction and fat accumulation, improving insulin sensitivity by modulating appetite and fat metabolism (63). Acupuncture, originating from China, was increasingly utilized worldwide for the treatment of diseases related to IR (64, 65). Acupuncture has the ability to correct metabolic dysregulation (65), lower hyperglycemia (66), improve body weight (67), suppress appetite (68), reduce lipids (69), exert anti-inflammatory effects (70), improve changes in sympathetic nervous system activity (71), and address insulin signaling deficits (72), as well as enhance insulin sensitivity (73).

## 5 Advantages and shortcomings

As far as we knew, this was the first time to conduct a comprehensive analysis of research in the field of diabetes and insulin resistance had been conducted, with special attention to its trends and hot spots, which would be very useful for scholars and researchers interested in this topic. Firstly, given the authority and comprehensiveness of the WOSCC, we only searched this database and may have overlooked the important value and information in other important database literature (e.g., PubMed, Scopus, Google Scholar, CrossRef). Secondly, we only included papers primarily written in English in our analysis, which may have resulted in a significant omission of excellent literature published in other languages. Finally, due to the continuous updates of the database and publication time, some recently published excellent papers with potential value may not have been fully researched and cited, which may lead to underestimation of the value and quality of the latest publications in bibliometric analysis results.

## 6 Conclusion

Using bibliometric methods, it was discovered that research in the fields of diabetes and IR was receiving increasing attention, with trends and hotspots being summarized into the following three key points. The association between IR, diabetes, and obesity has emerged as a popular research topic. Cardiovascular diseases secondary to diabetes and IR constitute another focal point of interest among researchers. As the core pathological change in diabetes, IR improvement was the primary therapeutic target in diabetes management. In the future, further in-depth research on the relationship between diabetes and IR, as well as its underlying mechanisms, held significant importance for the treatment and prevention of these diseases.

## Data Availability

The original contributions presented in the study are included in the article/supplementary material, further inquiries can be directed to the corresponding authors.
